# Passive Sensors for Long Duration Internet of Things Networks

**DOI:** 10.3390/s17102268

**Published:** 2017-10-03

**Authors:** Felisberto Pereira, Ricardo Correia, Nuno Borges Carvalho

**Affiliations:** Instituto de Telecomunicações, Departamento de Electrónica Telecomunicações e Informática, Universidade de Aveiro, Aveiro 3810-193, Portugal; rjoao@ua.pt (R.C.); nbcarvalho@ua.pt (N.B.C.)

**Keywords:** Wireless Sensor Networks, Radio Frequency Identification, passive sensor, low power sensor, backscatter communication, Wireless Power Transmission

## Abstract

In this work, three different concepts are used to develop a fully passive sensor that is capable of measuring different types of data. The sensor was supplied by Wireless Power Transmission (WPT). Communication between the sensor and reader is established by a backscatter, and to ensure minimum energy consumption, low power techniques are used. In a simplistic way, the process starts by the transmission of two different waves by the reader to the sensor, one of which is used in power transmission and the other of which is used to communicate. Once the sensor is powered, the monitoring process starts. From the monitoring state, results from after processing are used to modulate the incoming wave, which is the information that is sent back from the reader to the tag. This new combination of technologies enables the possibility of using sensors without any cables or batteries to operate 340 cm from the reader. The developed prototype measures acceleration and temperature. However, it is scalable. This system enables a new generation of passive Internet of Things (IoT) devices.

## 1. Introduction

**Figure 1:**
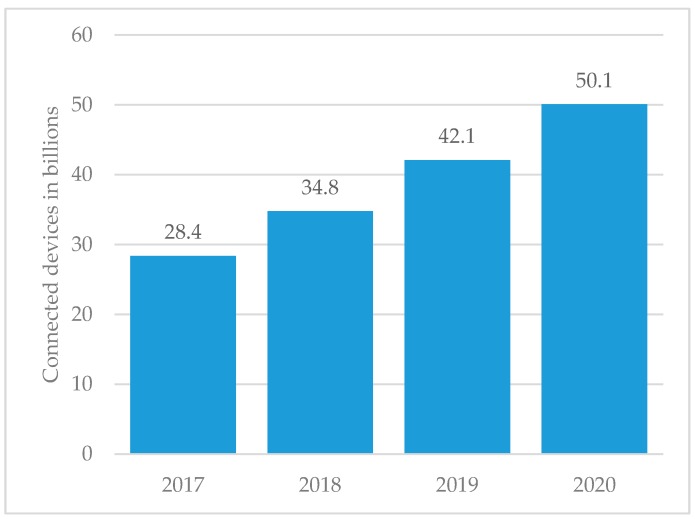
Internet of Things (IoT): Number of connected devices worldwide [8].

The concept of Wireless Sensor Networks (WSNs) has for some years attracted great academic and industrial attention. The advances in semiconductor, networking, and material science technologies are driving intelligent and low-cost devices to a large-scale paradigm, working autonomously with sensing, computation, and communication capabilities. The applications can range from environmental sensing and wearable biometric monitoring to security and structural monitoring, which perhaps realize the Internet of Things (IoT) concept [1,2,3]. WSN scalability is dependent on reduced costs and long-term deployment without battery replacement. The urgency of this topic is evident because in the present year of 2017, the predictions point to 28.8 billion IoT devices, a number that is expected to almost double in three years, as seen in Figure 1.

To address these constraints, systems have emerged that utilize communication by means of reflected Radio Frequency (RF) power [1]. The most popular system of this technology is Radio Frequency Identification (RFID), which was designed to allow for automatic identification. Its first application was a simple system that could detect the presence or absence of a tag. Although providing effective anti-theft measures continues to be one of the major applications of RFID, many other applications have appeared during the years. Currently, RFID plays a major function in the way people live it is essential in most objects, animals, or people [4,5].

Passive or battery-free RFID tags are an attractive option for WSNs. However, these tags are often application-specific fixed-function devices that can extract all of the power that they need from the carrier wave sent by the reader, as in passive tags used as barcodes alternatives. The devices used in WSNs are configurable, and they have computational power, requiring more power than normal RFID tags [6]. Semi-passive tags were created in an attempt to solve the power difficulties. Semi-passive tags have their own batteries. However, they are only used to power logic operations and not uplink communications [7]. The use of tags powered with batteries allowed enhancements in the Interrogation Zone (IZ): the carrier wave, which does not provide energy to the tag, can be completely reflected. Another method that is used to achieve longer distances between the tag and reader is the separation between carrier emitter and reader. In a monostatic approach, the carrier emitter and reader are located in the same device. To deliver maximum power to the tag, the carrier emitter is located near the tag, meaning that the reader is also close to the emitter. In a bistatic architecture, the carrier emitter and tags are in different locations. The carrier emitter is near the tag, but the reader can be located at a greater distance.

Reference [9] describes research involving a battery-powered tag and bistatic topology. It presents the results of a distance of 130 m between the tag and reader, with 4 m between he carrier emitter and tag. The carrier was transmitting a Continuous Wave (CW) with a power of 13 dBm. The same battery-powered tag and bistatic architecture is proposed in reference [10]. However, coherent detection and channel coding were used to improve the results to 150 m between the tag and reader and 10 m between the carrier emitter and tag. Although the results obtained by battery-powered tags are excellent in terms of distance, there are limitations: the battery cost is considerable and battery replacement can be a problem. The alternatives to battery-powered tags are tags with energy harvesting systems. Reference [11] proposed a system with photovoltaic cells to power tags, and in reference [12], the power source is motion. However, in the latter case, there are ambient power constraints, both on where one can place sensors and when they can be used [13].

To overcome the battery and energy harvesting restrictions, the alternative is to use energy transmission to supply the tag with power. The first idea on this subject, which concerned the transmission of energy from space down to Earth, was suggested by Peter Glaser (1969) in his project Solar Power Satellite. The idea was to place solar cells in space, and then send a microwave beam down to Earth. The wave would be received by a rectenna that would convert the radiation into Direct Current (DC) power [14]. Although the first idea for Wireless Power Transmission (WPT) focused on high power, the major developments in this field have been occurring in low power transmission.

In [15], a solution using inductive WPT and Ultra High Frequency (UHF) RFID was presented. The work proves that combining the inductive WPT with the UHF RFID increments the tag sensitivity in 21 dB, which increases the IZ. However, the use of inductive WPT requires proximity between the tag and the power source.

Regarding electromagnetic WPT, a comparison between different rectifier topologies and different stage levels was presented in [16]. The obtained results show a high dependence between the received power and the most efficient topology. A structure with a two-tone signal at 1.8 GHz and 2.4 GHz was shown in [17]. The results present a voltage output 20% higher in average when comparing with a single-tone input.

The work in reference [18] begins with a reader that is configured to transmit power in CW. After rectification, the power charges the storage capacitor to 5.5 V. The storage capacitor, once charged, powers the tag that performs sensing and communication, reflecting the carrier wave. This entire process occurs at a distance of 1 m between the reader and tag. The same principle is used in reference [6].

Different from the previous solutions, this work presents a solution using dual band wireless power and data transfer. One frequency is exclusively used to transmit energy to the sensor, and the other is fully dedicated to communication through backscatter. The technology was previously introduced without the use of any sensing nodes in reference [19]. A similar approach, using different frequencies for transmitted energy and communication was presented in [20]. In that work, for each frequency that is used a differentiated circuit for the RF-DC conversion. In the work presented in this manuscript, the entire process is done in a single circuit. The use of one single circuit reduces the number of components, which means lower losses, and allows for a more compact solution, which make a very reliable solution for IoT applications. It is important to refer that this work is a proof of concept regarding the combination of different techniques, upper layers like communication protocols are not taken into account.

This document is organized as follows. In the subsequent Section 2 and Section 3, the WPT and backscatter techniques are described and the methods used to combine both techniques are demonstrated. Section 4 explores the sensing and processing elements, first by outlining the components used and second by presenting the software iterations. All of the measurements and results are provided in Section 5 where the step-by-step process is explained and some considerations are also presented. Finally, Section 6 discusses the main conclusions of this work.

## 2. WPT and Backscatter Techniques

In recent years, the increasing use of IoT sensors has allowed smart objects to provide major industries with the vital data that they need to track inventory, manage machines, increase efficiency, save costs, and even save lives. In this context, in which billions of connected objects are expected to be placed all over the world, frequent battery maintenance of wireless nodes is undesirable or even impossible. In these scenarios, passive-backscatter radios will play a crucial role due to their low cost, low complexity, and battery-free operation. Wireless power transmission technology also plays an important role in providing continuous power for these backscatter radios. To implement the combination of these two technologies, the approach used in reference [21] was followed. In this approach, two different transmitter frequencies are used, one to supply the sensor and the other to communicate via backscatter.

**Figure 2:**
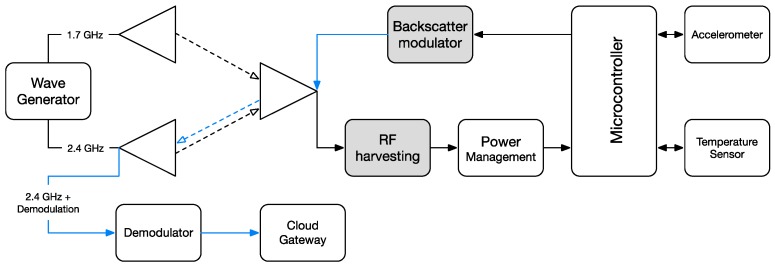
Block diagram of the implemented system.

Figure 2 shows a block diagram of the proposed combined technologies. Two main blocks are presented. The first, the RF harvesting circuit, is responsible for converting RF signals into DC energy to supply the microcontroller and modulate the information acquired by the sensors. The modulation is provided by switching the transistor ON and OFF, which is part of the backscatter modulator block. This process is known as Modulated Scattering Technique (MST) [22,23,24,25], resulting in this case in an Amplitude Shift Keying (ASK) modulation.

## 3. RF Front End Design and Optimization

**Figure 3:**
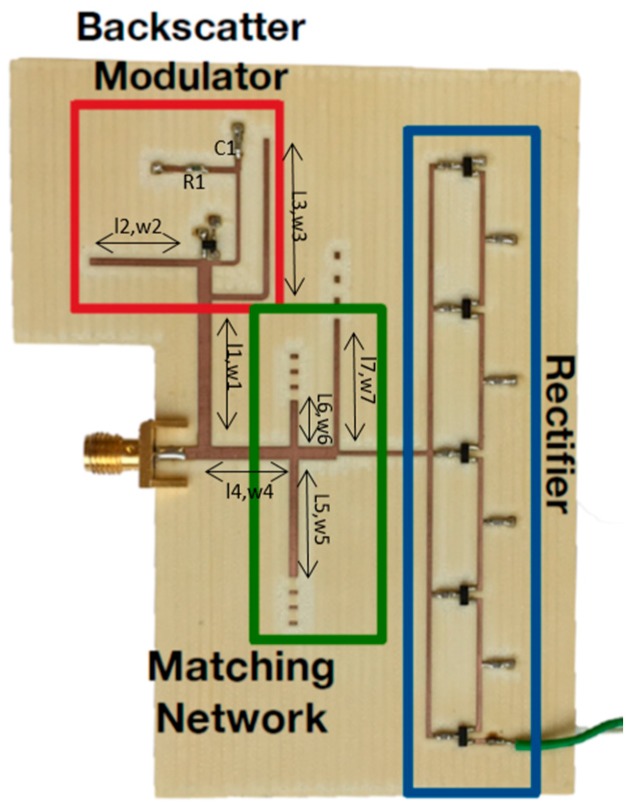
Photograph of implemented system with backscatter modulator combined with Wireless Power Transmission (WPT). Element values are L1 = 21.2 mm, W1 = 1.87 mm, L2 = 15.1 mm, W2 = 1.0 mm, L3 = 21.9 mm, W3 = 0.8 mm, L4 = 11.3 mm, W4 = 1.87 mm, L5 = 17.1 mm, W5 = 1.2 mm, L6 = 6.7 mm, W6 = 1.1 mm, L7 = 18.6 mm, W7 = 0.7 mm, R1 = 50 Ω, and C1 = 47 pF. Substrate for the transmission lines is Astra MT77, thickness = 0.762 mm, $\varepsilon_{r}$ = 3.0, tan δ = 0.0017.

The RF circuit is shown in Figure 3. It is divided into three main sections: the backscatter modulator, which is composed of a switch transistor that modulates the impedance of the antenna; the matching network, which was designed to provide backscatter load modulation at one frequency and continuous flow of WPT at other frequencies; and, the five-stage Dickson multiplier, which allows for RF-DC conversion to provide sufficient DC power to supply the microcontroller.

The main differences from the works presented in references [19,26] consist of the design and the optimization of the matching network to provide enough DC power to supply the digital processing unit. Once all of the lower frequency circuits within the sensor node were designed, the sleep current consumption that resulted from the sum of all of the quiescent currents was taken into consideration to model the load for the energy harvester, which was designed to mimic the real load that the harvester sees while the sensor node is sleeping. Out of all of the circuitry downstream from the output of the energy harvester, only the large storage capacitor and Schottky diode that precedes it were included into the simulation model of the load. All of the other circuits were replaced by an ideal current source that was placed in parallel with the storage capacitor with a value set equal to the estimated maximum sleep current consumption (that is, 5 μA). A harmonic balance simulation was performed in Advanced Design System (ADS) to tune the matching network for the maximum conversion efficiency and DC output with the ideal current. In references [19,26], simulations were performed for a fixed load value, without any consideration of the digital processing unit consumption.

To provide both backscatter communication and wireless power transfer, a Large Signal S-Parameter (LSSP) simulation was performed with the objectives of matching the circuit in both states of the transistor at 1.7 GHz, matching the system at 2.4 GHz in one state and mismatching at the other state. This change of the antenna’s impedance at 2.4 GHz provides the backscatter communication.

Regarding the RF transmission, three patch antennas were used. Two of them were connected to the wave generator matched at 1.7 GHz and 2.4 GHz, respectively, and the third connected to the sensor matched at both frequencies (dual-band). The patch antennas were chosen due to their high directivity.

## 4. Sensing and Processing Elements

The sensing part of the system is composed of a temperature sensor and an accelerometer, both of which were chosen carefully to have low power consumption and reliable measurements.

Texas Instruments’ LM94021 was the temperature sensor that was selected. In addition to its low power consumption (12 μA) and precise calibration, the sensor has a simple calibration process and temperature range from −50 °C to 150 °C [27]. Since the measurements performed during the tests were at ambient temperatures, the sensor was calibrated to operate between 0 °C and 40 °C, allowing for a better output resolution.

The second sensor was added to prove that the system can work with multiple sensors. Accelerometers were chosen due to their low power consumption, human interaction factor, and a wide range of possible applications. The accelerometer chosen was Analog Devices’ ADXL362, which consumes 1.8 μA at 100 Hz in operation mode and 10 nA in standby mode [28].

To acquire and process all of the data that come from the sensors, a processing unit is necessary. The chosen microcontroller was MSP430F2132 from Texas Instruments (Dallas, TX, USA). This microcontroller only consumes 2 μA in active mode and 0.3 μA in sleep mode. It also contains I/O ports, an Analog to Digital Converter (ADC), timers, and communications protocols. The only feature missing for the microcontroller with regard to the final application was a Digital to Analog Converter (DAC) unit. This unit was added to the system as an external component [29].

**Figure 4:**
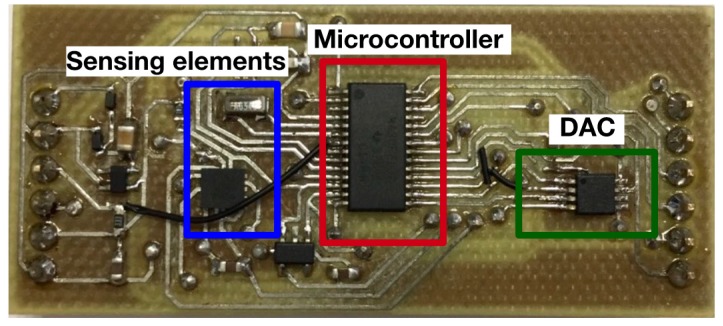
Developed Printed Circuit Board (PCB).

The sensing and processing elements were supplied by the RF-DC converter. The voltage delivery from the RF-DC converter depends on the distance from the reader as well as many other factors, such as wave reflections. Different levels of voltage create different behaviors in the components, and stable conditions are important for performing sensing activities. To solve this problem, a voltage regulator circuit was placed between the RF-DC circuit and sensing and processing units. The voltage regulator output was 1.8 V. In Figure 4, the PCB with the sensing and processing elements is shown.

**Figure 5:**
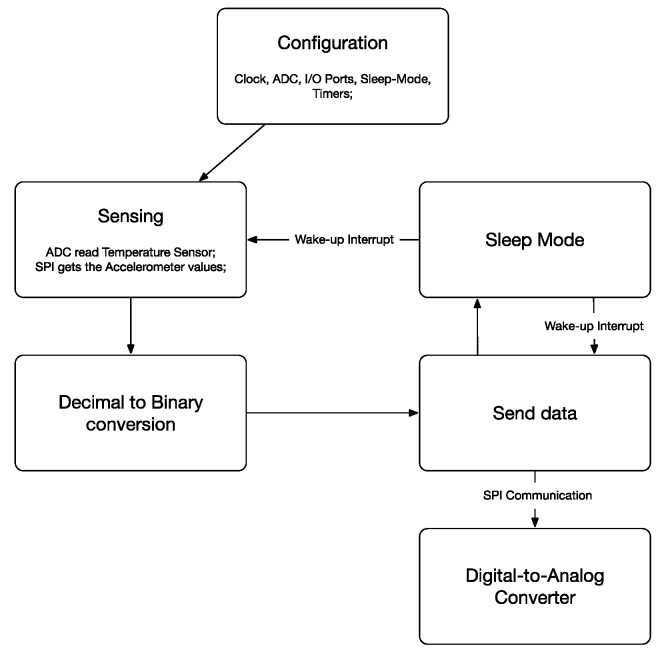
Code diagram.

As important as choosing low power components is in the software development, they should also be efficient to ensure that the components are only in active mode when they are actually needed. These techniques allow for a reduction of the system power consumption. The first software operation is the configuration of all of the elements, clocks, I/O ports, timers, ADC, DAC, and sleep modes. After configurations, the software processes the sensing block, reading the temperature and acceleration. The measured data need to be processed since misleading measurement errors can occur. A message is then produced to be delivered to the DAC. At this point, the DAC is responsible for controlling the system impedance by varying the voltage. When the message bit is a zero, the DAC output voltage is 0.0 V, which means that the system is adapted and, consequently, that there is no reflection. By contrast, when the message bit is one, the DAC output voltage is 0.6 V, which means that the impedance of the system is mismatched, which occurs with the reflection of the carrier wave. After sending each bit, the microcontroller enters sleep mode for a short period of time. When it sends the complete message, the microcontroller enters a longer period of sleep. Figure 5 illustrates the process described.

All of the message and sleep periods are defined by a simple variable on the code. The same is done with the output voltage delivered by the DAC. This gives the system the ability to easily modify the parameters for different applications. The message frame is composed of a total of 11 bits—3 bits to identify the tag ID, 2 bits to identify the sensor, 4 bits for the measurement, and 2 bits to detect errors in the message. The following Table 1 exemplifies the message frame with the coding resulting from the measured temperature:

The power consumption of the tag is defined by the bit period and sleep period. Long sleep periods result in low power consumption. The bit period is related to the frequency of the reflected wave, which defines the deviation of the modulated message from the carrier. Having a good deviation from the carrier wave is crucial to isolate the wave modulated by the tag from the carrier wave. Tests were conducted to optimize the periods. Periods of 366 μs for the bit period and 6.312 ms for the sleep period were established. The tag average consumption was 59 μA.

## 5. Measurements and Results

**Figure 6:**
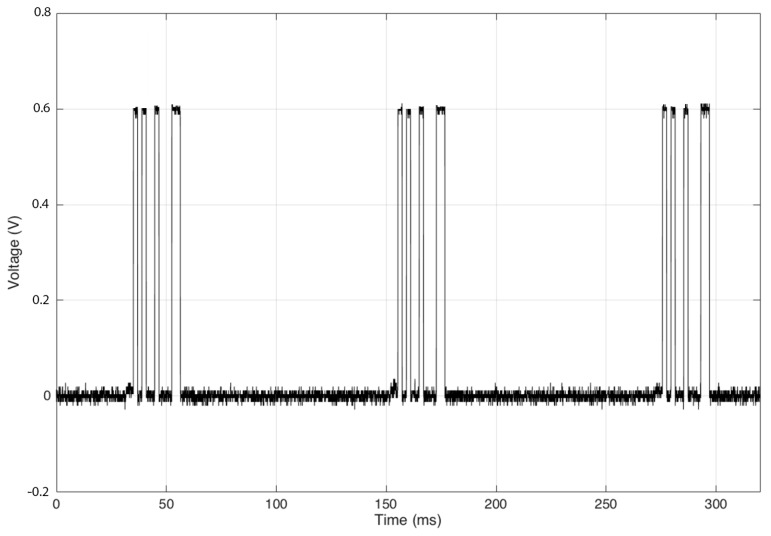
Message with tag ID, sensor ID, measured temperature, and correction bits.

The first measurements were to ensure the correct operation of the digital logic. Figure 6 shows the DAC output with the code “101-00-1000-11” that corresponds to a temperature between 17.5 °C and 20 °C.

As discussed previously, the goal of this work is to develop a passive tag. However, to perform and evaluate measurements, a reader is needed.

The reader is composed of a Rohde & Schwarz SMW200A Vector Signal Generator to generate two signals at different frequencies, one dual band patch antenna and a Rohde & Schwarz FSP Spectrum Analyzer (Munich, Germany) to analyze the signal modulated and reflected by the tag. The tag has a dual band antenna, a board with an RF-DC circuit, and another board with the processing and sensing elements. The RF-DC circuit and processing sensing elements are not on the same board to allow for specific tests for each approach. Figure 6 shows the measurements setup.

**Figure 7:**
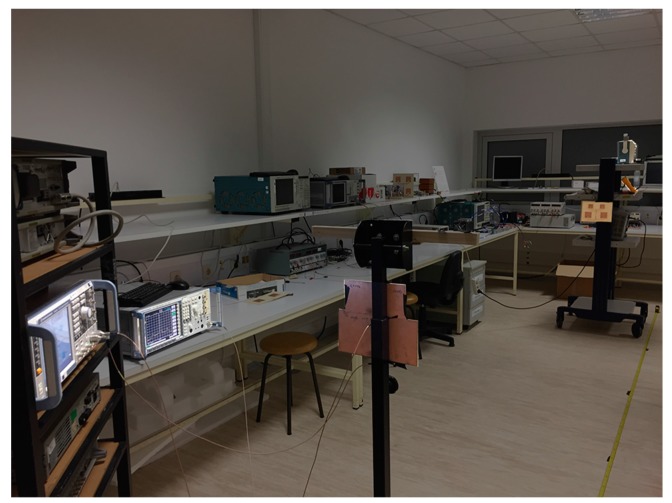
Measurements setup.

The first measurement was taken to determine the optimal frequencies for system operation. The S11 parameter of the antennas and DC output voltage of the rectifier were measured. In Figure 7, it is observed that there is a very good relationship between the most efficient point on the RF-DC and antenna adaptation. The optimal frequencies for both are on the same points. Based on the results, the frequency chosen for WPT was 1.720 GHz and for performing backscatter communication was 2.405 GHz.

The following measurements are used to evaluate the input power and output voltage after the RF-DC circuit under different test conditions. There are three parameters that changed during the tests: load, tag adaptation, and power delivery. No load means that there is no load attached to the RF-DC circuit, and a load occurs when the sensing and processing board are attached to the RF-DC circuit. Backscatter absorbing is the state at which the DAC output voltage is 0.0 V, meaning that the circuit is at its most adapted point (maximum absorption). In backscatter reflection, the DAC output is 0.6 V, corresponding to a mismatch of the system impedance (maximum reflection). The power delivery to the system varies from −20 dBm to 0 dBm.

**Figure 8:**
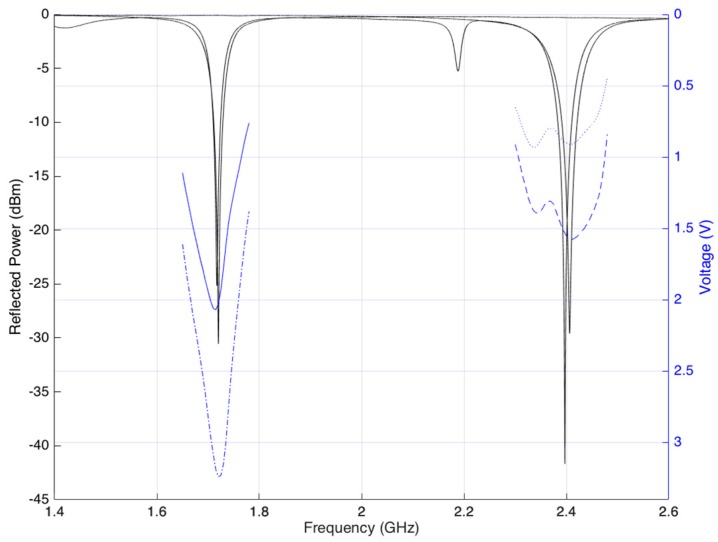
Optimal operational frequency.

The results are shown in Figure 8. It can be concluded that the frequency that is exclusively used for WPT is not affected by the backscatter absorption or the reflection state. The figure also shows that the wave with 2.405 GHz generates more DC voltage when the system is adapted than when it is not. Another aspect is that there is a decrease of voltage when a load is present. As is known, maintaining the delivered power and increasing the current makes the voltage drop.

**Figure 9:**
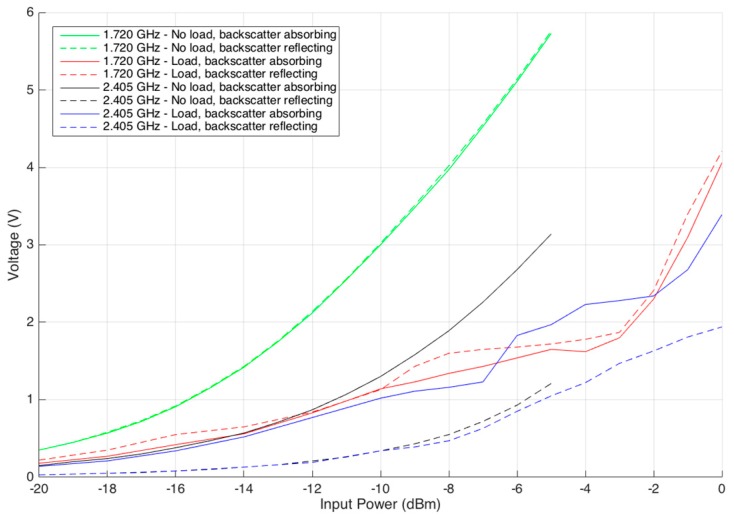
Input power vs. output voltage.

To better understand the reliability of the WPT technique, the reader and tag were placed at a distance of 300 cm from each other. During the test, the power transmitted by the reader was varied from 0 dBm to 30 dBm. By measuring the power received by the tag at both frequencies, its respective output voltage, and the tag state (ON or OFF), it is possible to obtain all of the information about the WPT process. Figure 9 presents the results.

Figure 9 shows that an increment in the transmitted power results in linear growth of the received power for both frequencies until the tag is OFF. The tag turns ON when the transmitted power is near 26 dBm. At this point, the tag starts performing the backscatter process, changing between reflecting and absorbing states. The ON state of the tag is reflected on the receiver power, at 2.405 GHz, where a decrease is visible on the growing line after that point.

In addition to the previous test for understanding the characteristics of WPT, another test was conducted to measure the maximum distance at which the reader can supply the tag. The maximum transmitted power, 30 dBm, was defined, and the maximum distance of 430 cm between reader and tag was measured. In this point it is important to refer that European Telecommunications Standards Institute (ETSI) legislates the amount of power that can be transmitted. For an UHF RFID the legal limitation is 33 dBm Effective radiated power (ERP). The value of 30 dBm was used due to laboratory limitations.

**Figure 10:**
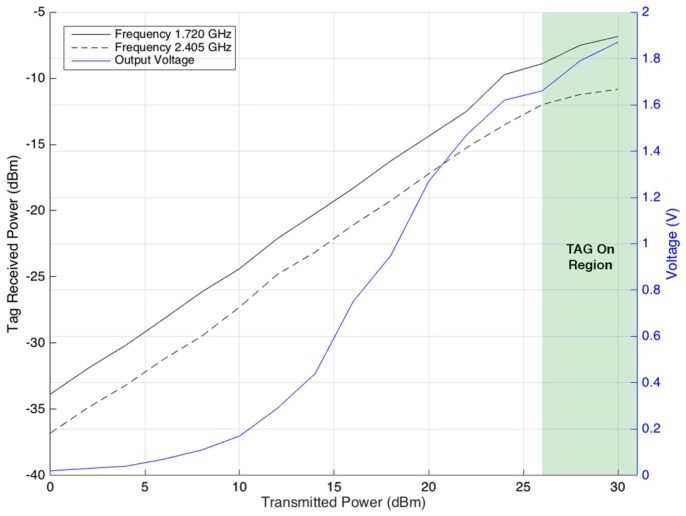
WPT at distance of 300 cm.

Even though the maximum distance at which the reader can supply the tag is 430 cm, this does not mean that the complete system can work at that distance. For this system to work, the tag needs to modulate the signal and reflect it with enough power so that it can be received by the reader. To understand all of the behaviors, new measurements were made. The received power at the tag at both frequencies, power reflected by the tag, and power received by the reader were measured. These measurements were made by varying the transmitted power between 10 dBm and 30 dBm, and the distance between reader and tag was recorded. All of the results are presented in Figure 10.

**Figure 11:**
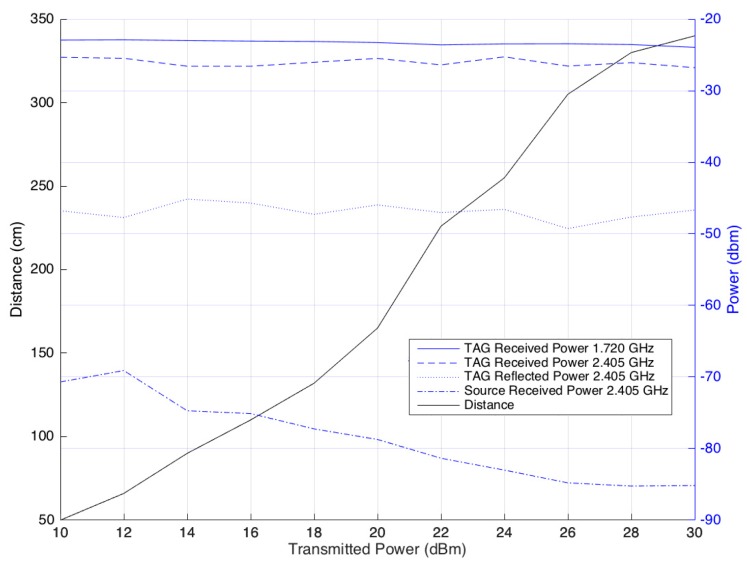
Transmitted power vs. maximum distance.

The power received by the tag does not suffer large variations, which is natural once the minimum received power to turn the tag ON is constant. Since the power received is constant and reflection always occurs the same way from the tag, it is logical that the reflected power has a value that does not have large variations. However, since the tag always reflects the same amount of power and the distance between reader and tag increases, the power received by the reader decreases. At a distance of 340 cm between reader and tag, the reflected power that arrives at the tag is approximately −85 dBm. Values below this threshold do not allow the reader to demodulate the signal, meaning that communication between the tag and reader is not possible at a distance further than 340 cm, as can be seen in Figure 11.

Considering the results and to better understand them, the free space loss was calculated based on measurements. At the maximum distance, 340 cm, the sensor is reflecting −46.67 dBm and the reader is receiving −85.17 dBm, these values were measured using a coupler (Marki, Morgan Hill, CA, USA, CBR16-0012) to differentiate incident from reflected waves. The total amount of losses is:

(1) $${\mathrm{Power}}_{\mathrm{received}}={\;\mathrm{Power}}_{\mathrm{reflected}}+{\mathrm{Antennas}}_{\mathrm{Gain}}-\mathrm{Losses}\;\langle =\rangle \;\langle =\rangle \mathrm{Losses}=-{\;\mathrm{Power}}_{\mathrm{received}}+{\mathrm{Power}}_{\mathrm{reflected}}+{\mathrm{Antennas}}_{\mathrm{Gain}}=46.5\;\mathrm{dB}$$

Despite this, we can not use the Friis formula in this scenario, due to multipath effects and other imperilments we use it to show that the measured results are not out of scope. Considering the same distance obtained in the experiments, the theoretical losses were calculated:

(2) $$\mathrm{Losses}=\;{\left(\frac{\lambda }{4\pi R}\right)}^{2}=\;42.68\;\mathrm{dB}$$

This validates the measured results.

## 6. Conclusions

In this paper, a passive tag with sensing and processing elements was presented. The developed tag has two sensing elements: temperature and acceleration. It is important to note that the microcontroller has the capability to incorporate a greater number and different types of sensors. The aim is to use the tag in multiple scenarios, especially situations that do not allow for a physical connection, maintenance and access. Agriculture, space, and smart house applications are good examples for which this technology could be used. In agriculture, sensors need to be underground to measure soil conditions. This scenario is not favorable for battery replacement. In space applications, the monitoring system is usually connected by a large amount of cables. Removing the cables would decrease the ship weight and consequently fuel consumption. In smart house applications, the sensors could be embedded at different locations, making different types of measurements.

One of the contributions of this paper is the measurement and testing of the technology, which combines WPT and backscatter techniques. This technology enables the use of one frequency exclusively for power transmission and another for communication, which allows for greater distances between the reader and tag. Development of the sensing and processing elements to ensure maximum power efficiency is an important addition. The obtained results show a very good distance at which it is possible for the sensor to communicate its measurements after they are handled by its processing unit.

## Figures and Tables

**Table 1:** Corresponding code from the measured temperature.

Temperature (Celsius)	Code (bits)
0–2.5 °C	101-00-0001-11
2.5–5 °C	101-00-0010-11
5–7.5 °C	101-00-0011-01
(…)	(…)
37.5–40 °C	101-00-1111-01
